# Is there any correlation between spectral CT imaging parameters and PD‐L1 expression of lung adenocarcinoma?

**DOI:** 10.1111/1759-7714.13273

**Published:** 2019-12-05

**Authors:** Mai‐Lin Chen, An‐Hui Shi, Xiao‐ting Li, Yi‐Yuan Wei, Li‐Ping Qi, Ying‐Shi Sun

**Affiliations:** ^1^ Key Laboratory of Carcinogenesis and Translational Research (Ministry of Education/Beijing), Radiology of Department Peking University Cancer Hospital & Institute Beijing China; ^2^ Key Laboratory of Carcinogenesis and Translational Research (Ministry of Education/Beijing), Radiotherapy of Department Peking University Cancer Hospital & Institute Beijing China

**Keywords:** Computed tomography, lung adenocarcinoma, PD‐L1

## Abstract

**Background:**

The aim of this study was to explore whether spectral computed tomography (CT) imaging parameters are associated with PD‐L1 expression of lung adenocarcinoma.

**Methods:**

Spectral CT imaging parameters (iodine concentrations [IC] of lesion in arterial phase [ICLa] and venous phase [ICLv], normalized IC [NICa/NICv]‐normalized to the IC in the aorta, slope of the spectral HU curve [λHUa/λHUv] and enhanced monochromatic CT number [CT40keVa/v, CT70keVa/v] on 40 and 70 keV images) were analyzed in 34 prospectively enrolled lung adenocarcinoma patients with common molecular pathological markers including PD‐L1 expression detected with immunohistochemistry. Patients were divided into two groups: positive PD‐L1 expression and negative PD‐L1 expression groups. Two‐sample Mann‐Whitney U test was used to test the difference of spectral CT imaging parameters between the two groups.

**Results:**

The CT40keVa (127.03 ± 37.92 vs*.* −54.69 ± 262.04), CT40keVv (124.39 ± 34.71 vs. −45.73 ± 238.97), CT70keVa (49.56 ± 11.76 vs. −136.51 ± 237.08) and CT70keVv (46.13 ± 15.81 vs. −133.10 ± 230.72) parameters in the positive PD‐L1 expression group of lung adenocarcinoma were significantly higher than the negative PD‐L1 expression group (all *P* < 0.05). There was no difference detected in IC, NIC and λHU of the arterial and venous phases between both groups (all *P* > 0.05).

**Conclusion:**

CT40keVa, CT40keVv, CT70keVa and CT70keVv were increased in positive PD‐L1 expression. These parameters may be used to distinguish the PD‐L1 expression state of lung adenocarcinoma.

## Introduction

Primary lung cancer is the leading cause of morbidity and mortality both in China and worldwide.^1^, ^2^ Lung adenocarcinoma is the most common histopathological type of lung cancer. With the development of molecular pathology, rapid progress has been made in the diagnosis and treatment of lung adenocarcinoma. In addition to conventional chemotherapy, targeted therapies targeting epidermal growth factor receptor (EGFR) and receptor tyrosine kinase (ALK), which block mutant targets of lung adenocarcinoma, have become the first‐line treatment of choice.^3^, ^4^ At the same time, programmed death ligand‐1 (PD‐L1) plays an important role in tumorigenesis and development, and promotes the wide application of immunotherapy in lung cancer.^5^, ^6^

At present, the detection of lung cancer‐related mutant genes and PD‐L1 expression is mainly through invasive biopsy, some of which are expensive, which limits its wide clinical use.

Recently, the correlation between CT signs of lung adenocarcinoma and common gene mutations of EGFR and ALK has been studied.^7^, ^8^, ^9^, ^10^, ^11^, ^12^, ^13^, ^14^ It has been reported that EGFR mutation is correlated with CT signs, such as ground‐glass density, burr, air bronchus sign, and ALK mutation is more common in the lobular margin. These signs may be helpful in predicting gene mutations in patients with advanced lung cancer without biopsy.^11^

Spectral CT imaging has been applied in several of the studies on pulmonary nodules. Most researchers believe that quantitative parameters of spectral CT imaging have certain advantages in the differential diagnosis of benign and malignant pulmonary nodules.^15^, ^16^, ^17^, ^18^, ^19^, ^20^, ^21^, ^22^, ^23^ There is no study testing the association between the spectral CT imaging parameters and the PD‐L1 expression in lung adenocarcinoma. Thus, this study was prospectively conducted to analyze whether spectral CT imaging parameters were associated with PD‐L1 expression of lung adenocarcinoma.

## Methods

### Patients

From January 2018 to August 2018, a total of 34 patients detected with immunohistochemistry of 67 lung adenocarcinomas (14 men, 20 women; age range, 45–81 years; mean age, 61.5 ± 7.5 years), were prospectively enrolled in the study. This research protocol was approved by the Medical Ethical Committee of Peking University Cancer Hospital & Institute and written informed consent was obtained from all patients in accordance with the guidelines of National Health Commission of the People's Republic of China. Patients were selected for investigation according to the following inclusion criteria: (i) presence of at least one solitary lung adenocarcinoma proved by pathology and (ii) no contraindications to the administration of iodinated contrast material. Patients without PD‐L1 testing were excluded from this study.

### CT examinations

CT examinations were performed with two‐phase enhanced CT scanning using spectral imaging mode on a Revolution Xtream CT scanner (GE Healthcare, WI, USA). Patients were injected with 40/50 mL (≤70 kg bodyweight, 40 mL; >70 kg bodyweight, 50 mL) Iopromide (Ultravist 300; Bayer Schering Pharma AG, Guangzhou, Guangdong, China) at a flow rate of 5/6 mL/second (≤70 kg bodyweight, 5 mL/second; >70 kg bodyweight, 6 mL/second), followed by the 30 mL saline solution at the same injection rate. With a scan delay of 30 and 90 seconds after the start of contrast injection, a GSI examination of the entire chest was performed during arterial phase (AP) and portal venous phase (VP), respectively. There were no serious injection complications or issues in this study. Acquisition parameters were helical tube rotation time 0.6 seconds, helical pitch 0.985, tube current of 600 mA, 512 × 512 pixel matrix, SFOV 500 mm and collimation 40 mm, slice thickness of 5 mm, slice gap of 5 mm. Contiguous axial images (2.5 and 1.25 mm thickness) at a default monochromatic energy level of 40 and 70 KeV were then reconstructed with a soft tissue kernel (standard) with GSI data file. CT dose index (CTDI_vol_) for GSI acquisition was 4.73 mGy.

### Quantitative analysis of spectral CT images

All data were processed and analyzed by GSI Volume Viewer software package at AW4.7 work station (GE HealthCare, USA). Monochromatic and material decomposition images were performed to analyze the quantitative measurements by a chest radiologist with 10 years experience. During data analysis, the radiologist amplified the display field of view to 15 or 20 cm in each lesion imaged. The region of interest (ROI) was selected as large as possible at the maximum section of the lesion carefully avoiding calcification, liquefaction, or necrosis, away from pulmonary vessels and bronchi, and as large as possible to reduce noise (50 pixels). All measurements were repeated three times at three contiguous imaging levels and average values calculated to ensure consistency.

Spectral curve image, iodine‐based material decomposition images, and monochromatic images obtained at the energy level of 40 and 70 keV in both arterial phase (a) and venous phase (v) were reconstructed from the spectral CT acquisition for analysis. In the iodine density image derived from the iodine/water based material decomposition image, the iodine concentration of lesions (ICLa/ ICLv) in double‐phase enhanced scan were measured. The iodine concentration in the aorta descendens or subclavian artery (ICA) were also measured in the same slice. The normalized iodine concentration (NICa and NICv), which is the ratio of iodine concentration in lesion and aorta descendens (NIC = ICL/ICA), were calculated. These iodine concentration parameters ICLa, ICLv, ICAa, ICAv, NICa and NICv were calculated in the arterial and venous phase, respectively. The slope of spectral HU curve (λHU) was assessed only 40–70 keV region by the equations λHU = (CT40 keV−CT70 keV) HU/(70–40) based on previous studies. λHUa = (CT40 keVa−CT70 keVa) HU/(70–40) and λHUv = (CT40 keVv−CT70 keVv) HU/(70–40) were calculated.

### Pathological evaluation ‐ immunohistochemical determination of PD‐L1

Pathological specimens were routinely fixed in 10% formalin, paraffin‐embedded. Tissue sections were cut at 4 μm thickness including the largest cut surface of the tumor, and stained with hematoxylin and eosin (H&E). Pathological diagnoses were made by two experienced lung pathologists, based on the new WHO histological classification of lung tumors in 2015,^24^ as atypical adenomatous hyperplasia (AAH), adenocarcinoma in situ (AIS), minimally invasive adenocarcinoma (MIA) and invasive adenocarcinoma (IAC).

Immunostaining was performed using the standard streptavidin‐perosidase (SP142) technique with the antibodies for PD‐L1. A Leica automatic staining machine was used for immunohistochemical staining. Tumor proportion score (TPS) was counted. TPS >1% was positive and TPS >50% indicated a high expression of PD‐L1.^5^, ^6^, ^24^

Immunohistochemical EnVision two‐step staining was used to detect antibodies P40, TTF‐1, NapsiA and ki‐67.

### Statistical analysis

Data were transformed using a Box‐Cox power transformation by Statistics 12 (Dell Inc., Round Rock, TX, USA), to minimize the influence of extreme values or non‐normal distributions. Parameters were expressed as mean ± SD and tested for normal distribution using Kolmogorov‐Smirnov test. The two‐sample Mann‐Whitney test was used to compare the difference of spectral CT quantitative parameters between positive PD‐L1 expression and negative PD‐L1 expression groups. A *P*‐value less than 0.05 indicated significance. SPSS was used to perform the statistical analysis (version 18.0; Chicago, IL).

## Results

A total of 34 patients with PD‐L1 test results were enrolled into the study. The results indicated that there was one patient (2.9%) with minimally invasive adenocarcinoma (MIA) (2.9%) and 33 with invasive adenocarcinomas (IACs) (97.1%). Of these, 15 were solid cases and 19 ground‐glass opacities, which presented as predominant lepidic growth in nine cases, predominant acinar growth in nine cases, solid growth in two cases, papillary growth in two cases, micropapillary growth in one case, infiltrating mucinous adenocarcinoma in one case, and mixed of two growth in 10 cases. All cases were confirmed by pathology. PD‐L1 expression was positively detected in eight cases (23.5%), and PD‐L1 expression was negative in 26 cases (76.5%).

**Figure 1:**
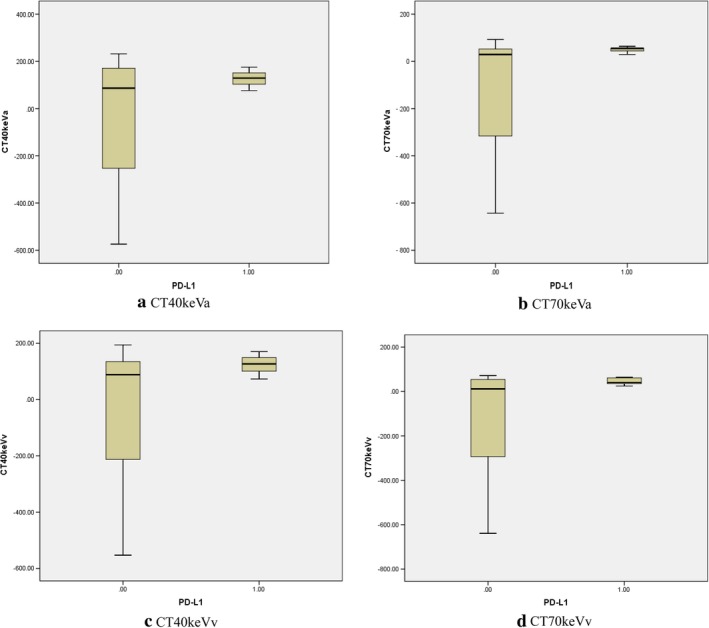
Comparing the spectral CT imaging parameters between positive and negative groups, the values of (**a**) CT40 keVa, (**b**) CT40 keVv, (**c**) CT70 keVa and (**d**) CT70 keVv in the positive group were higher than those in the negative group.

**Figure 2:**
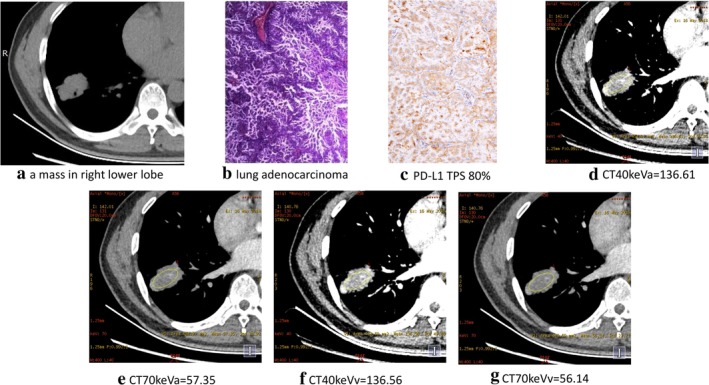
A 45‐year‐old male with right lower lung adenocarcinoma had 80% PD‐L1 TPS, on (**a**) conventional CT, (**b**) pathological section, (**c**) PD‐L1 expression, (**d**) CT40keVa, (**e**) CT70keVa, (**f**) CT40keVv and (**g**) CT70keVv.

In the positive PD‐L1 expression and negative PD‐L1 expression groups, raw variables for differentiating positive PD‐L1 expression from negative PD‐L1 expression are shown in Table 1, Figures 1 and 2 versus 3. The CT40keVa, CT40keVv, CT70keVa and CT70keVv were increased in the positive PD‐L1 expression group compared with the negative PD‐L1 expression group. (CT40keVa: 127.03 ± 37.92 vs. −54.69 ± 262.04, *P* = 0.005; CT40keVv: 124.39 ± 34.71 vs. −45.73 ± 238.97, *P* = 0.004; CT70keVa: 49.56 ± 11.76 vs. −136.51 ± 237.08, *P* = 0.002; CT70keVv: 46.13 ± 15.81 vs. −133.10 ± 230.72, *P* = 0.002, as seen in Figures 2 versus 3.

**Table 1:** Difference between positive PD‐L1 expression and negative PD‐L1 expression

Parameters	Positive (*n* = 8)	Negative (*n* = 26)	*P*‐value
CT40keVa (HU)	127.03 ± 37.92	−54.69 ± 262.04	0.005
CT70keVa (HU)	49.56 ± 11.76	−136.51 ± 237.08	0.002
ICLa (mg/mL)	13.38 ± 5.41	14.25 ± 7.35	0.778
NICa (mg/mL)	0.39 ± 0.19	0.42 ± 0.31	0.792
λHUa	2.58 ± 1.00	2.96 ± 1.48	0.543
CT40keVv (HU)	124.39 ± 34.71	−45.73 ± 238.97	0.004
CT70keVv (HU)	46.13 ± 1581	−133.10 ± 230.72	0.002
ICLv (mg/mL)	13.77 ± 3.56	14.09 ± 4.16	0.857
NICv (mg/mL)	0.65 ± 0.18	0.78 ± 0.69	0.635
λHUv	2.61 ± 0.69	2.98 ± 1.07	0.399

**Figure 3:**
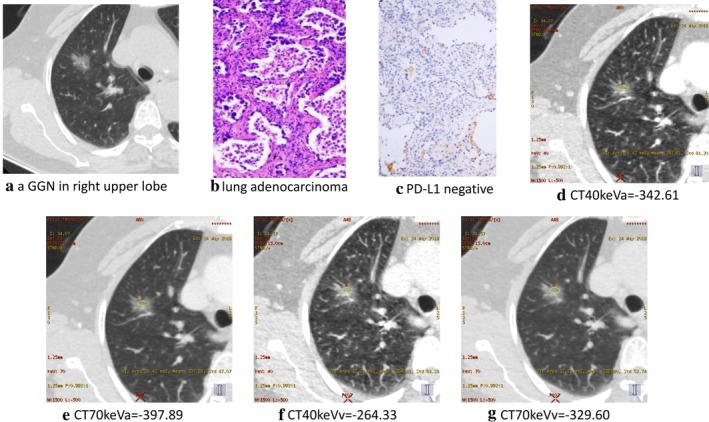
A 69‐year‐old female with lung adenocarcinoma had negative PD‐L1 expression on (**a**) conventional CT, (**b**) pathological section, (**c**) PD‐L1 expression, (**d**) CT40keVa, (**e**) CT70keVa, (**f**) CT40keVv and (**g**) CT70keVv.

IC, NIC and λHU in both arterial and venous phases in the positive PD‐L1 expression group had no statistically significant differences compared to the negative PD‐L1 expression group, and all were *P* > 0.05.

## Discussion

Currently, the choice of first‐line treatment for advanced non‐small cell lung cancer (NSCLC) depends on the presence of genetic aberrations, such as mutations of epidermal growth factor receptor (EGFR) and translocations of anaplastic lymphoma kinase (ALK). However, only 10% to 20% of patients with NSCLC have these actionable mutations.^25^ For the remaining patients, treatment options are limited to platinum‐based cytotoxic chemotherapy, with a response rate ranging between 15% and 30%.^26^, ^27^ The recent development of immunotherapy which targets the programmed death‐1/programmed death‐ligand 1 (PD‐L1) axis has been approved for first‐line treatment as well as second‐line treatments of NSCLC in many countries. It alone has significantly improved overall survival (OS) compared with chemotherapy for first‐line NSCLC with PD‐L1 TPS ≥50%, 20%, ≥1%.^5^, ^6^, ^24^, ^28^ The higher the PD‐L1 expression, the better the objective response rate and median survival time. Therefore, PD‐L1 expression has been recorded as a good predictive biomarker, although it is variable across clinical trials and influenced by the tumor microenvironment, based on immunohistochemistry assays.

At present, using immunohistochemistry (IHC) staining in formalin‐fixed paraffin‐embedded tissue samples is the most popular method to assess PD‐L1 expression. However, there is no unified standard to detect PD‐L1 staining because there are different staining techniques and antibodies. Scoring methods and cutoff values to define positive expression are also different in individual studies which make it difficult to reach a compatible consensus.

Radiographic assessment as a noninvasive and quantitative method could bring practical clinical benefit in the development of predictive markers before immunotherapy. Similarly, traditional chest CT features have also been extensively studied as radiologic markers for predicting therapeutic effect and survival time of lung adenocarcinomas. In addition, several studies have been conducted on the relationship between traditional chest CT features and gene mutations of EGFR, ALK in lung adenocarcinomas.^7^, ^8^, ^9^, ^10^, ^11^, ^12^, ^13^, ^14^ As far as we know, there has been no research on the correlation of spectral CT imaging parameters and PD‐L1 expression status to date.

In this study, and as seen in Table 1 and Figure. 1, there was a significant difference in spectral CT imaging parameters between the positive PD‐L1 expression and negative groups, and the spectral CT imaging parameters (CT40keVa, CT40keVv, CT70keVa and CT70keVv) were increased in positive PD‐L1 expression. We believe that the difference in spectral CT imaging parameters with different PD‐L1 expression status could be biological and pathophysiological more than just statistical and mathematical. The high expression of PD‐L1 was involved in tumor immune escape and promotes the occurrence and development of tumor, with more nutrition and blood supply at the same time. The increased spectral CT imaging parameters in positive PD‐L1 expression also indirectly reflected this transition. Just as in previous studies, molecular imaging biomarkers radiolabeled with ^89^Zr, ^64^Cu, ^68^Ga or ^111^In^29^, ^30^, ^31^, ^32^ in immuno‐PET/CT can noninvasively monitor numbers and localization of intratumoral, systemic alterations of immune cells during treatments, which may help clinicians to understand the dynamics of immunotherapeutic mechanisms and clarify possible methods for detecting immunotherapy responses. In the same way that radiolabeled imaging biomarkers reflect tumor hemodynamic information, perhaps the spectral CT imaging parameters may help to detect PD‐L1 expression and quantitatively reflect the dynamic changes of PD‐L1 expression before, after, or at different checkpoint of treatment, and this may be need to be explored and verified in future research. Additionally, the recent radiomic‐based predictive approach, especially CT‐derived predictive model, may be conducive to anticipate PD‐L1 expression status,^33^, ^34^ particularly in NSCLC patients.^33^ Radiomic features from the tumor and its periphery features can provide information on both the tumor and its microenvironment. The spectral CT imaging parameters based on internal hemodynamic information of lung tumor may also be suitable to evaluate the changes of tumor microenvironment, such as the expression of PD‐L1.

There were several limitations in this study. First, the number of patients was relatively small, and the PD‐L1‐positive cases were proportionally lower, which probably results in selection bias; however, it is epidemiologically consistent with the incidence of lung cancer with PD‐L1 expression. It means that our results should be further validated in a large number of cases. Second, the biological behavior of different subtypes of lung adenocarcinoma varies greatly, and their spectral CT imaging parameters and PD‐L1 expression may be different. Third, the majority of selected cases in this study were elderly people with a mean age of 61.5 years, and younger patients should be included in future studies. Fourth, monochromatic CT number was measured only enhanced monochromatic spectral CT imaging at the energy level 40 and 70 keV based on previous studies,^15^, ^16^, ^17^, ^18^, ^19^, ^20^, ^21^, ^22^ ignoring the other keV selection. Fifth, quantifying microvessel densities previously described in a previous study^35^ were not included in this study.

In conclusion, spectral CT imaging parameters, CT40keVa, CT40keVv, CT70keVa and CT70keVv were significantly increased when PD‐L1 expression was positive. These parameters may be used to distinguish the PD‐L1 expression status of lung adenocarcinoma.

## Disclosure

The author(s) have no relevant conflicts of interest to disclose.
